# Calcium-activated potassium channels mediated blood-brain tumor barrier opening in a rat metastatic brain tumor model

**DOI:** 10.1186/1476-4598-6-22

**Published:** 2007-03-14

**Authors:** Jinwei Hu, Xiangpeng Yuan, MinHee K Ko, Dali Yin, Manuel R Sacapano, Xiao Wang, Bindu M Konda, Andres Espinoza, Ksenia Prosolovich, John M Ong, Dwain Irvin, Keith L Black

**Affiliations:** 1 Department of Neurosurgery, Maxine Dunitz Neurosurgical Institute, Cedars-Sinai Medical Center, Los Angeles, CA 90048. USA

## Abstract

**Background:**

The blood-brain tumor barrier (BTB) impedes the delivery of therapeutic agents to brain tumors. While adequate delivery of drugs occurs in systemic tumors, the BTB limits delivery of anti-tumor agents into brain metastases.

**Results:**

In this study, we examined the function and regulation of calcium-activated potassium (K_Ca_) channels in a rat metastatic brain tumor model. We showed that intravenous infusion of NS1619, a K_Ca _channel agonist, and bradykinin selectively enhanced BTB permeability in brain tumors, but not in normal brain. Iberiotoxin, a K_Ca _channel antagonist, significantly attenuated NS1619-induced BTB permeability increase. We found K_Ca _channels and bradykinin type 2 receptors (B2R) expressed in cultured human metastatic brain tumor cells (CRL-5904, non-small cell lung cancer, metastasized to brain), human brain microvessel endothelial cells (HBMEC) and human lung cancer brain metastasis tissues. Potentiometric assays demonstrated the activity of K_Ca _channels in metastatic brain tumor cells and HBMEC. Furthermore, we detected higher expression of K_Ca _channels in the metastatic brain tumor tissue and tumor capillary endothelia as compared to normal brain tissue. Co-culture of metastatic brain tumor cells and brain microvessel endothelial cells showed an upregulation of K_Ca _channels, which may contribute to the overexpression of K_Ca _channels in tumor microvessels and selectivity of BTB opening.

**Conclusion:**

These findings suggest that K_Ca _channels in metastatic brain tumors may serve as an effective target for biochemical modulation of BTB permeability to enhance selective delivery of chemotherapeutic drugs to metastatic brain tumors.

## Background

The blood-brain barrier (BBB), formed by the capillary endothelial cells surrounded by astrocytes, protects the brain, but it also poses an obstacle for the delivery of therapeutic molecules into the brain. Microvessels supplying brain tumors retain some characteristics of the BBB and form a blood-brain tumor barrier (BTB). While adequate delivery of chemotherapeutic drugs has been achieved in systemic tumors, the BTB limits such delivery to brain metastases. Therefore, understanding the biochemical modulation of BBB and BTB is critical for developing strategies to deliver therapeutic agents into metastatic brain tumors.

During the past decade, various strategies have been used to deliver therapeutic drugs selectively to brain tumors and injured brain, including, biodegradable polymers implanted into the tumor cavity [1], convection-enhanced delivery [2,3], and BBB/BTB disruption [4,5]. Our laboratory has focused on pharmacologic modulations to increase BTB permeability and increase delivery of therapeutic drugs selectively to brain tumors with little or no drug delivery to normal brain tissue [6-9]. This strategy exploits the function of certain vasomodulators that play a key role in modulation of BBB/BTB permeability. It has been demonstrated that bradykinin [10], leukotriene (LTC_4_) [11-13], nitric oxide (NO) [14], c-GMP [8], and potassium channel agonists [15,16] can selectively increase capillary permeability in primary brain tumors, while leaving normal brain unaffected. These findings have already been translated into clinical studies to increase drug delivery selectively to tumor tissue in brain tumor patients [7,17-19]. Modulation of critical molecules involved in selectively increasing BTB permeability could lead to the development of effective strategy to increase chemotherapy delivery to brain tumors.

Large conductance calcium-activated potassium (K_Ca_) channels are a unique class of ion channel coupling intracellular chemical and electrical signaling. These channels give rise to outwardly rectifying potassium currents and respond not only to changes in membrane voltage, but also to changes in intracellular calcium. Recent studies suggest that K_Ca _channel expression levels correlate positively with the malignancy grade of glioma [20]. K_Ca _channels are also present in cerebral blood vessels, where they regulate cerebral blood vessel tone [21] and, probably, BBB/BTB permeability [15,22]. Evidence from several studies further indicate that K_Ca _channels play an important role in vasodilation when it is mediated by bradykinin [23,24], NO donors [25], and cyclic GMP [26]. In response to the binding of bradykinin to its type 2 receptors (B2R), intracellular Ca^2+ ^is increased either by mobilization of Ca^2+ ^from internal sites and influx [27] or by NO production from NO synthase activation [14]. The increase in intracellular Ca^2+ ^level activates K_Ca _channels and alters the membrane potential of cells [28]. Furthermore, previous studies have also shown that bradykinin-induced K_Ca _channel activation in endothelial cells is potentiated by NS1619, a selective K_Ca _channel agonist [29], and attenuated by a highly selective inhibitor, iberiotoxin (IBTX) [29-31]. We previously demonstrated that K_Ca _channels are overexpressed in primary brain tumors and tumor microvessels, and such channels respond to NS1619, which selectively increases BTB permeability. The accelerated formation of pinocytotic vesicles appears to be the cellular mechanism by which K_Ca _channels mediate increases in BTB permeability [15]. Moreover, in a rat brain tumor model, we showed that the B2R expression level on brain tumors directly correlates with bradykinin-induced BTB permeability increases [32]. Co-infusion of carboplatin with either NS1619 or a bradykinin analog, RMP-7, led to enhanced survival in intracranial tumor-bearing rats and brain tumor patients [17-19,22,33]. These data indicate that K_Ca _channels serve as a convergence point in the modulation of BTB permeability in primary brain tumors.

Brain metastasis is a frequent complication in patients suffering from lung and breast cancer, and a significant cause of morbidity and mortality. Brain metastases are found in approximately 10% of lung cancer patients at the time of diagnosis, and up to 40% of all patients develop brain metastases during the course of their disease [34]. The prognosis of brain metastases from lung cancer is poor, with median survival of 4~5 month. Lung cancer cells that spread to the brain are generally sensitive to chemotherapeutic drug compared with primary brain tumor cells. The BTB, however, prevents the delivery of non-lipid-permeable chemotherapeutic drugs and monoclonal antibodies in sufficient amounts to achieve a therapeutic benefit [35], especially in early stage of brain metastases. Although metastatic brain tumors have ten times more than the incidence of primary brain tumors in the United States, the role and regulation of K_Ca _channels in metastatic brain tumors to selectively open BTB have not been elucidated. As new therapeutic agents are developed which effectively treat primary tumors, an efficient delivery of these agents selectively to metastatic brain tumors across the BTB will significantly improve treatment efficacy. Here, we studied the role of K_Ca _channel activation in BTB permeability in a metastatic brain tumor model.

## Results

### K_Ca _channel mediates BTB permeability increase in a CRL-5904 metastatic brain tumor model

**Figure 1:**
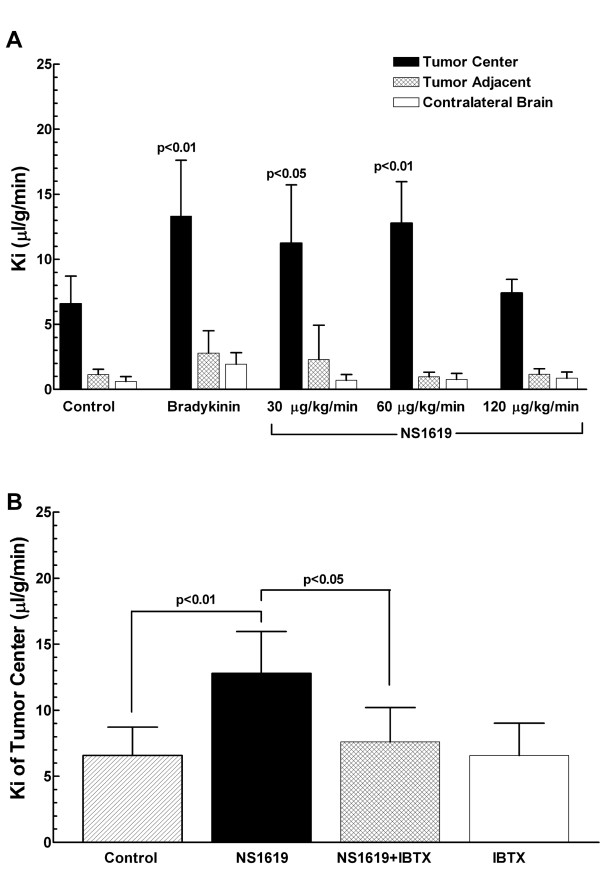
Regional *Ki *values in metastatic brain tumor-bearing animals. Quantitative increases in BTB permeability after biochemical modulation are shown in dose denpendent manner (A). Intravenous infusion of NS1619 (30 μg/kg/min; n = 5) significantly increased *Ki *in the tumor center compared with PBS control (*p *< 0.05). The optimal dose of NS1619 (60 μg/kg/min; n = 5) further increased *Ki *value (*p *< 0.01, NS1619 vs PBS). At higher dose (120 μg/kg/min; n = 6), however, no BTB permeability increased with infusion of NS1619. Intravenous infusion of BK (120 μg/kg/min; n = 3) also significantly increased *Ki *in the tumor center compared with control (*p *< 0.01). NS1619 (60 μg/kg/min)-induced BTB permeability increase was significantly attenuated by a specific K_Ca _channels antagonist, IBTX (1 μg/kg/min; n = 4), (*p *< 0.05, NS1619+IBTX vs NS1619). While, intravenous infusion of IBTX (n = 3) alone did not show any effect on BTB permeability (B).

To determine whether K_Ca _channels mediate BTB permeability in a metastatic brain tumor model, intracranial CRL-5904 tumor bearing-rats received intravenous infusion of NS1619 (0~120 μg/kg/min), bradykinin, IBTX, or PBS. The transport constant (*Ki*) was determined by radiotracer [^14^C] sucrose uptake in the tumor core, tumor-adjacent brain tissue, and contralateral brain tissue. The data were obtained from 3~6 rats for each group. Intravenous infusion of NS1619 at 30 μg/kg/min resulted in a significant increase of *Ki *values in the tumor center (11.24 ± 1.99 μl/g/min) as compared with PBS control (6.58 ± 0.75 μl/g/min; *p *< 0.05, NS1619 vs PBS). A higher concentration of NS1619 at 60 μg/kg/min further increased *Ki *values (12.77 ± 1.99 μl/g/min; *p *< 0.01, NS1619 vs PBS). While, increasing dose to120 μg/kg/min did not elicit any further *Ki *increase. Intravenous infusion of bradykinin (120 μg/kg/min) also significantly increased BTB permeability within the tumor, with average *Ki *values at 13.31 ± 2.48 μl/g/min(*p *< 0.01, bradykinin vs PBS). Furthermore, NS1619- and bradykinin- induced BTB permeability increases resulted in enhanced delivery of radiotracer [^14^C] sucrose to the tumor center without any increase to tumor adjacent brain tissue and contralateral normal brain (Figure 1A). In a separate experiment, we found that co-administration of a specific K_Ca _channel inhibitor, IBTX (1 μg/kg/min), blocked the increase of BTB permeability induced by NS1619 (*Ki *value of NS1619+IBTX is 7.58 ± 1.31 μl/g/min; NS1619+IBTX vs NS1619, *p *< 0.05). Intravenous infusion of IBTX alone did not show any effect on BTB permeability (Figure 1B). These data confirm that activation of K_Ca _channel selectively induces BTB opening in tumor tissue, but not tumor adjacent tissue or contralateral normal brain, in a metastatic brain tumor animal model.

### Expression of K_Ca _channels and B2R in CRL-5904 cells, HBMEC and human tumor tissue of lung cancer brain metastases

**Figure 2:**
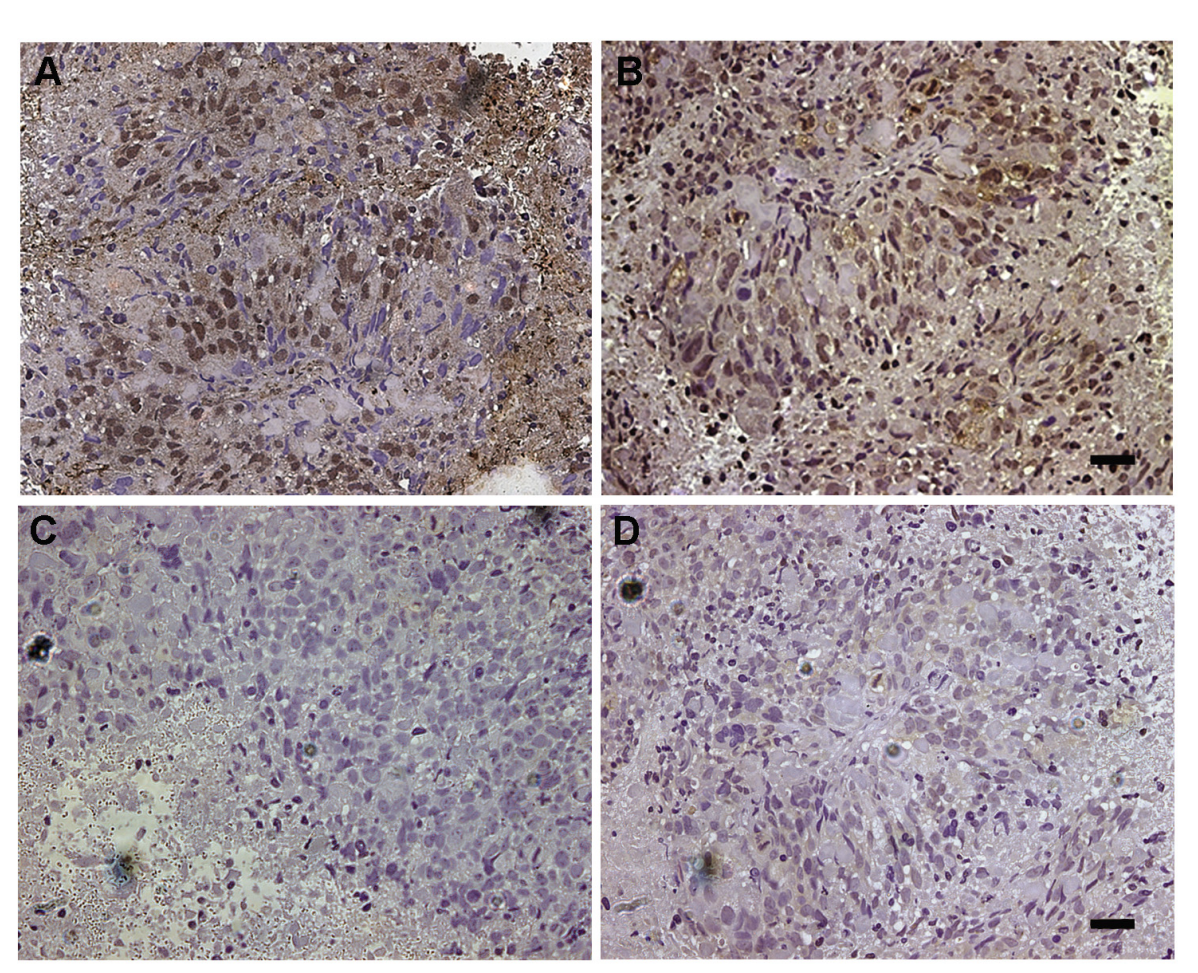
Expression of K_Ca _channels and B2R in the lung cancer metastatic brain tumor tissue from patients. K_Ca _channels (A) and B2R (B) expression were detected on the tumor cells in the tissue. Negative control experiments of K_Ca _channels (C) and B2R (D) did not show specific staining on the corresponded specimens by deleting of primary antibodies. Scale bar = 50 μm.

**Figure 3:**
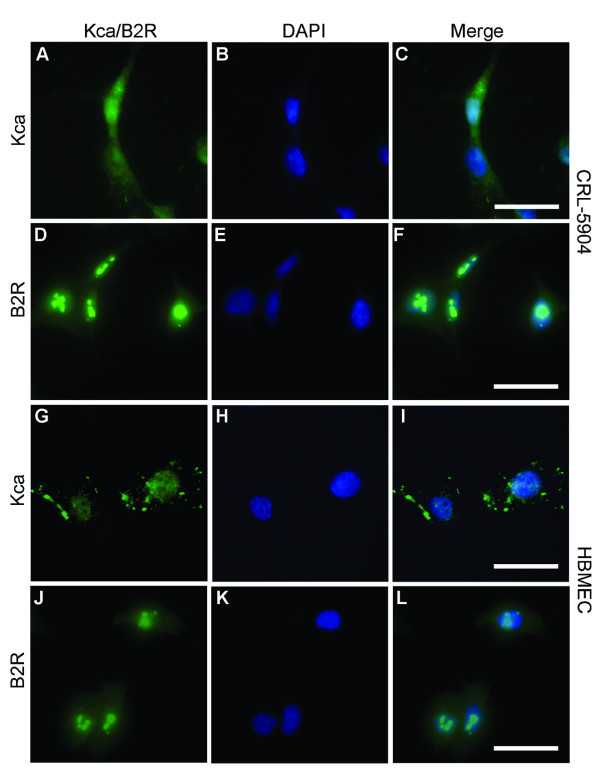
Expression of K_Ca _channels and B2R in CRL-5904 cells and HBMEC. K_Ca _channels (green) distributed in CRL-5904 cells (A~C) and HBMEC (G~I). The B2R (green) were expressed in CRL-5904 cells (D~F) and HBMEC (J~L). Cells were located by counterstaining with DAPI (blue, B, E, H, K) and merged images are also shown (C, F, I, L). Scale bar = 50 μm.

To examine whether K_Ca _channels were present in tumor tissue, immunostaining of paraffin-embedded tissue sections from lung cancer brain metastases patients were performed. The results demonstrated that K_Ca _channels (Figure 2A) and B2R (Figure 2B) expressed extensively in tumor masses and microvessels within the tumor. Negative control experiments of K_Ca _channels (Figure 2C) and B2R (Figure 2D) did not show specific staining on the corresponded specimens. Elevated mRNA level of K_Ca _channels was also detected in lung cancer brain metastases tissues from patients using real time PCR (data not shown). To further determine whether K_Ca _channels and B2Rs are present in metastatic brain tumor and endothelial cells, we examined their expression by immunocytochemistry. Fluorescence immunostaining showed robust K_Ca _channel expression in cultured CRL-5904 cells, which distributed on the cell membrane, cytoplasm and perinuclear components (Figure 3A~C). K_Ca _channels were also detected in HBMEC (Figure 3G~I), but the signal intensities were lower compared with that in CRL-5904 cells. B2R expression was detected in both CRL-5904 cells (Figure 3D~F) and HBMEC (Figure 3J~L) with a higher level of expression in CRL-5904 cells. These data illustrate the presence of K_Ca _channels in cultured metastatic brain tumor cells, endothelial cells, and most importantly, in human metastatic brain tumor tissue.

### Activity of functional K_Ca _channels in cultured CRL-5904 cells and HBMEC

**Figure 4:**
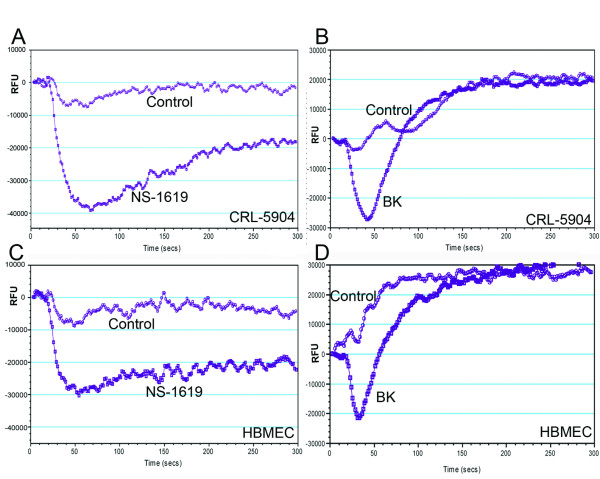
Measurement of functional K_Ca _channels activity in CRL-5904 cells and HBMEC. Membrane potential changes in relative fluorescence units (RFU) were detected during a 300-second response to 25 μM of NS1619 (A) and BK (B) respectively on the CRL-5904 cells. NS1619 and BK also elicited membrane potential changes on HBMEC (C, D). Addition of IBTX (20 nM) reversed the membrane potential to resting values.

Since the above data demonstrated the presence of K_Ca _channels in metastatic brain tumors and endothelial cells as well as metastatic brain tumor tissue, we determine whether the overexpressed K_Ca _channels were functional. Therefore we examined membrane potential activity of K_Ca_channels in cultured tumor and endothelial cells and by using a fluorescent dye-based potentiometric assay that measures changes in membrane potential. To activate K_Ca _channel, NS1619 (25 μM), a K_Ca _channel agonist, was added to the cells, and membrane potential changes were monitored for up to 300 seconds. Upon activation a decrease in membrane potential was observed in CRL-5904 cells (Figure 4A) and HBMEC (Figure 4C), an effect that lasted more than 300 seconds. Furthermore, we introduced bradykinin (25 μM) to the cultured cells and observed membrane potential changes in CRL-5904 cells (Figure 4B) and HBMEC (Figure 4D) that lasted for approximately 100 seconds. Moreover, both NS1619 and bradykinin elicited greater hyperpolarization on CRL-5904 cells compared with HBMEC. IBTX (20 nM), a K_Ca _channel antagonist, reversed the membrane potential changes on both cells caused by NS1619 and bradykinin. Furthermore, we found that NS1619 and bradykinin could induce a dose-dependent membrane potential change in CRL-5904 and HBMEC (data not shown). These data indicate that K_Ca _channels are functional on both CRL-5904 cells and HBMEC. The K_Ca _channels can be activated directly by NS1619 or indirectly through B2R signaling by bradykinin.

### Co-culture of metastatic brain tumor and endothelial cells increases K_Ca _Channel expression

**Figure 5:**
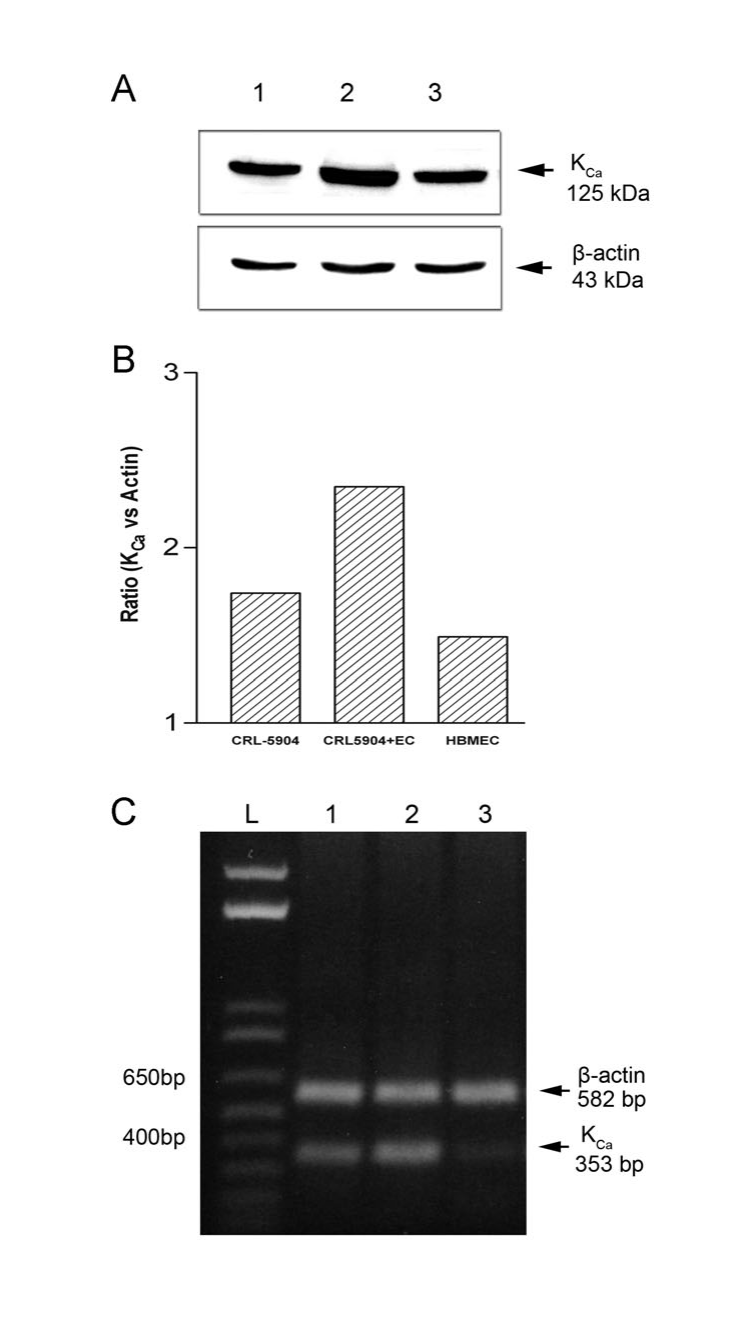
Modulation of K_Ca _channel expression in co-cultured cells (CRL-5904 cells and HBMEC). Western blot analysis for K_Ca _channel expression on cultured cells. Lane1, CRL-5904 cells; Lane 2, CRL-5904 and HBMEC co-culture; Lane 3, HBMEC (A). Semi-quantitative analyses of the protein bands were normalized by internal control, β-actin (B). mRNA transcription levels of different cell cultures were determined by RT-PCR. L: 1 kb ladder; Lane1, CRL-5904 cells; Lane 2, CRL-5904 and HBMEC co-culture; Lane 3, HBMEC (C).

We further investigated whether K_Ca _channels expression is modulated by the interaction of metastatic brain tumor cells and endothelial cells. CRL-5904 cells were co-cultured with HBMEC, and protein and mRNA levels of K_Ca _channels were examined subsequently. K_Ca _channel were overexpressed in CRL-5904/HBMEC co-cultures compared to single cultures of either CRL-5904 cells or HBMEC by western blot assay (Figure 5A). Image quantification analysis showed an approximately 30% increase of K_Ca_channel expression in co-culture of CRL-5904/HBMEC compared to individual cultures by normalized to β-actin as an internal control (Figure 5B). Also, individual cultures of CRL-5904 tumor cells had higher K_Ca _channel expression than HBMEC. RT-PCR analysis also showed an increase in K_Ca_channel mRNA levels in co-culture cells compared to individual cultures (Figure 5C). These data suggest that co-culture of metastatic tumor and brain endothelial cells results in upregulation of K_Ca _channel.

### Immuno-colocalization of K_Ca _channel expression in a CRL5904 metastatic brain tumor animal model and human lung cancer brain metastases tissue

**Figure 6:**
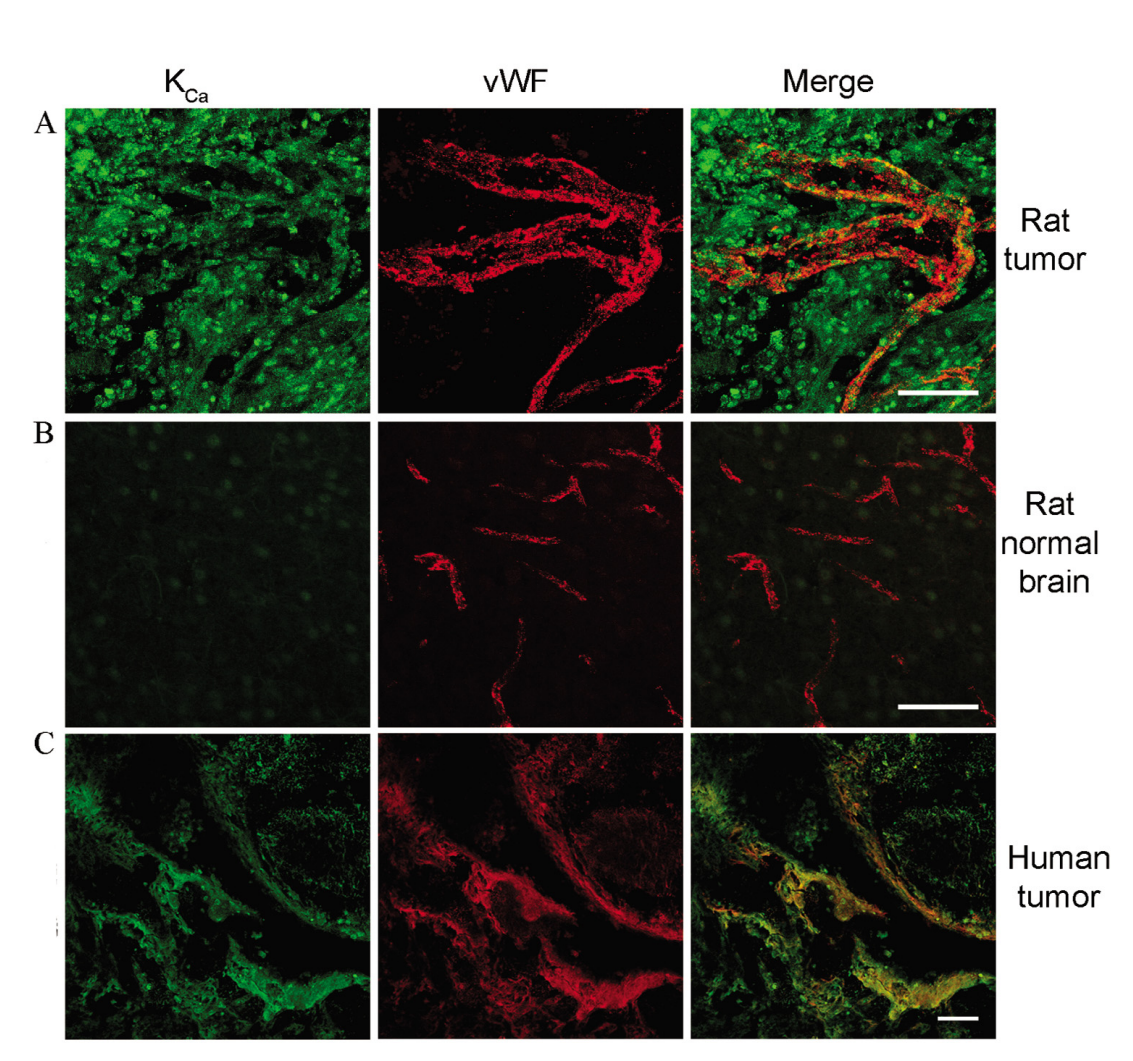
Localization of K_Ca _channels in brain tumor tissues from xenogenic rat model and patients of lung cancer brain metastases. K_Ca _channels (green) were localized in the endothelial cells of capillaries (red) in the tumor mass (A). In the contralateral side of brain, much less of K_Ca _channel was detected in normal brain. (B). K_Ca _channels are co-localized with von Willebrand factor, a marker of endothelial cells, in human tumor tissue of lung cancer brain metastases (C). Scale bar = 250 μm.

Since K_Ca _channel modulators can selectively increase BTB permeability without affecting normal brain, we wanted to know whether K_Ca _channels were differentially expressed within the tumor mass compared with normal brain tissue. To address this question, we examined K_Ca _channel and endothelial cell marker von Willebrand factor (vWF) expression in CRL-5904 tumors and human lung cancer brain metastases tissue. An abundance of K_Ca_channel expression (green) was detected within the tumor mass with robust microvessel formation (red) within the tumor area of CRL5904 tumor (Figure 6A). In contrast, there was less K_Ca _channel expression in normal brain tissue adjacent to the tumor mass as well as contralateral normal brain (Figure 6B). More importantly, colocalization of K_Ca _channels with vWF within microvessels was observed only within the tumor mass and not in normal brain. These results demonstrate elevated expression of K_Ca_channels on endothelial cells of capillaries within the tumor mass. Immunostaining of human tissue from lung cancer brain metastases also revealed that K_Ca _channel expression was colocalized with vWF expression in tumor capillary endothelia (Figure 6C). These results strongly suggest that the selective BTB permeability increase induced by modulation of K_Ca _channel in the metastatic brain tumor model is likely due to the overexpresion of K_Ca _channels on tumor cells and tumor capillary endothelia.

## Discussion

We have studied the presence of K_Ca _channels and B2R in primary brain tumors, however, their occurrence and function in metastatic brain tumors remained to be investigated. In this study, we detected high level expression of K_Ca _channels in CRL-5904 tumor and brain endothelial cells, which is consistent with previous studies showing K_Ca _channels expression in RG2 glioma and endothelial cells [15]. Other investigators have also demonstrated that the expression level of K_Ca _channels correlates with the malignancy grade of glioma in human [36]. Therefore, these data suggest there is an intimate association between K_Ca _channel expression and brain tumor development, which remains to be fully investigated. Additionally, we detected the presence of B2R in CRL-5904 tumor and endothelial cells. Liu *et al *also showed that B2R are expressed in cultured RG2, C6 and 9L glioma cells, more interestingly, the expression levels of B2R in tumor cells was directly correlated with the increase of BTB permeability induced by bradykinin in a rat glioma model [32]. Thus, the presence of K_Ca_channels or B2R in metastatic brain tumor cells or HBMEC may play a functional role in BTB permeability of metastatic brain tumors.

We examined K_Ca _channel activity on metastatic tumor cells and capillary endothelial cells using a membrane potential assay, which is well-correlated with the patch-clamp method, used to measure changes in membrane potential (FLIPR application note, Molecular devices, CA). We found that NS1619 and bradykinin elicited greater hyperpolarization effects on CRL-5904 than on HBMEC. These findings may reflect a higher level of expression for K_Ca _channels on metastatic tumor cells as compared to endothelial cells. Importantly, the membrane potential change induced by NS1619 lasted 3 times longer than that induced by bradykinin. These data further support the finding, from cellular level, that NS1619-elicited increases in BTB permeability in a glioma model last up to 60 minutes compared to the transient effect of bradykinin, which lasts for about 15~20 minutes [15], partially due to B2R internalization [37]. The current data illustrates that the presence of K_Ca _channel are functional in metastatic brain tumor and endothelial cells. Similar to our findings, Reiser *et al *demonstrated that bradykinin can directly activate K_Ca _channels in rat glioma cells [38]. Other studies have shown that bradykinin can activate K_Ca _channels through a NO-cGMP signalling pathway [39]. Hence, our present study indicates that bradykinin-activated downstream signals, such as activation of K_Ca_channels, may be modulated to induce membrane potential changes on brain metastatic tumor and endothelial cells.

The presence of functional K_Ca _channels in metastatic brain tumor and brain endothelial cells suggests that biochemical modulation of K_Ca_channels could play an important role in therapeutic BTB opening. We further investigated whether the K_Ca _channels agonist, NS1619 and bradykinin could selectively enhance BTB permeability in a metastatic brain tumor xenograft model. These results showed that intravenous infusion of NS1619 yielded a two-fold increase the unidirectional transport of a radiotracer into metastatic brain tumors; similar to bradykinin-induced BTB permeability increase in metastatic brain tumor-bearing rats. Our previous studies have demonstrated that higher doses of intravenous bradykinin are required to increase BTB permeability compared to intracarotid infusion of bradykinin, reflecting the influence of the first pass effect with intracarotid delivery [15]. In a glioma model, it has been reported that the effects of bradykinin on BTB permeability mediated by B2R resulted in enhanced drug delivery to glioma [40]; and this effect could be attenuated by coinfusion with IBTX [15]. In this metastatic brain tumor model, we further demonstrate the presence of B2R and confirm that the bradykinin effect on permeability is mediated via K_Ca _channels. Consistent with previous studies [41], current data confirms the selective increase of BTB permeability in brain metastatic tumors but not normal brain tissue. These results suggest that biochemical modulation of K_Ca _channel induces a selective BTB opening in metastatic brain tumor.

Confocal images showed K_Ca _channels overexpression in tumor tissue and tumor microvessels as compared with normal brain. More importantly, the tumor capillaries showed co-localization of K_Ca _channels and vWF in tumor area of CRL-5904 tumor and in human metastatic brain tumor tissue. To further study the interaction between tumor and endothelial cells, we co-cultured CRL-5904 metastatic brain tumors and brain endothelial cells. We show that mRNA expression of K_Ca _channels is upregulated in co-cultured cells compared to indivdual cultures. These data suggest that increased K_Ca _channel expression and their activity in tumor endothelial cells maybe due to the tumor micro-environment or cell-to-cell communication between tumor and microvessel endothelial cells. This is consistent with reports that brain tumor cells increase K_ATP _channel expression in endothelial cells[16].

These findings support a pivotal role for K_Ca _channels in BTB permeability regulation. Recently clinical study showed that trastuzumab, anti-HER2 antibody, while effective in treating tumors outside the brain, fails to treat brain metastases due to its inability to cross the blood brain tumor barrier [42]. Our research has shown that K_Ca_channel-mediated BTB permeability modulation could be a useful strategy to increase therapeutic agents, such as antibody-based therapies, delivery into metastatic brain tumors.

## Conclusion

We present evidence that activation of K_Ca _channels by a channel-specific agonist can selectively enhance BTB permeability in a metastatic brain tumor rat model. We show K_Ca _channel and B2R are highly expressed in brain metastatic tumor cells, endothelial cells and lung cancer brain metastatic tissue. The expression level is correlated with K_Ca _channel activity in these cells. In a metastatic brain tumor model, we demonstrate that NS1619 and bradykinin can selectively open BTB and significantly enhance the radiotracer delivery specifically to metastatic brain tumors. It is also demonstrated that K_Ca _channels expression can be upregulated in the co-cultures of tumor cells and endothelial cells, as well as in the microvessel endothelia of brain metastases tissue. K_Ca_channels may be exploited as specific target for selectively pharamacologic modulation of BTB to increase delivery of chemotherapeutic drugs to brain metastases.

## Methods

### Cell Culture

CRL-5904 cells (human non-small cell lung cancer; metastatic site: brain poorly differentiated carcinoma) and human brain microvessel endothelial cells (HBMEC) were obtained from the American tissue culture collection (ATCC, VA) and maintained in RPMI 1640 with 10% fetal bovine serum. Both cell lines were maintained in the common tissue culture condition. For co-culture of CRL-5904 cells with HBMEC, the same number of CRL-5904 cells and HBMEC were co-cultured in growth medium and allowed to achieve 90% confluence. Then, the co-culture and single cultures of cells were harvested for protein or RNA extraction.

### Membrane Potential Assay

The functional activity of K_Ca _channels in CRL-5904 cells and HBMEC was measured using the FLIPR Membrane Potential Assay Kit on a FLEXstation (Molecular Devices, Sunnyvale, CA) as described previously [15]. This kit provided a fast, simple and consistent mix-and read procedure. In brief, the cells were seeded in sterile, clear bottom, black 96-well plates (Corning Inc., MA) at density of 2 × 10^3 ^cells/well to achieve monolayer within 24 h. The monolayer cells were incubated with the membrane potential assay kits reagents for 30 min before loading the compounds. The anionic potentiometric dye that transverses between cells and extracellular solution in a membrane potential-dependent manner serves as an indicator of vasomodulator-induced voltage changes across the cell membrane. Dose response studies were performed with 0 to 50 μM NS1619 or bradykinin with or without IBTX (20 nM). The FLEXstation was set up using the following parameters: excitation 530 nm, emission 565 nm, and emission cut of 550 nm wavelengths. Observations and recordings were made for 300 seconds after adding the compounds. NS1619, bradykinin and IBTX were obtained from Sigma (St. Louis, MO).

### In Vivo BBB/BTB Permeability

All of the animals used were conducted in accordance with the Institutional Animal Care and Usage Committee in force at Cedars-Sinai Medical Center. A metastatic brain tumor xenograft model was established using athymic nude rats (180–200 g; Charles River Laboratories, Inc., MA) for BBB/BTB permeability studies. Athymic nude rats were anesthetized with i.p. ketamine and xylazine, and stereotactically implanted with CRL-5904 cells (2 × 10^5^) in 4 μl of 1.2% methylcellulose/PBS using a Hamilton syringe into the right striatum. The Coordinates were 3.4 mm lateral to bregma and 5.0 mm deep from dura. Ten days after tumor implantation, the femoral arteries of rats were cannulated to measure blood pressure and collect blood, and the femoral vein was also cannulated to administer the drugs and radiotracer. Body temperature was maintained at 37°C. Arterial blood gases, blood pressure and hematocrit were monitored. Animals with abnormal physiological parameters were eliminated from this study. In regional permeability studies, either intravenous drug or PBS was infused into the femoral vein at a rate of 66.7 μl/min for 15 minutes. Five minutes after the start of the intravenous infusion, 50 μCi/kg of the radiotracer [^14^C] sucrose was injected as an intravenous bolus. Arterial blood pressure was monitored throughout the experimental period with a blood pressure monitor (DigiMed, KY). The unilateral transport constant *Ki *(μl/g/min), which is an initial rate for blood-to-brain transfer of radiotracer, was calculated as described by Ohno et al[43]. The *Ki *was determined by radiotracer [^14^C] sucrose in the tumor core, tumor-adjacent brain tissue, and contralateral brain tissue using the quantitative autoradiographic (QAR) method as describe previously [44]. Quantitative analysis of the regional radioactivity was performed using a computer (Power Macintosh 7100) and Image 1.55 software (National Institutes of Health, Bethesda, MD). An optimum dose (120 μg/kg/min, i.v.) of bradykinin established previously was used for *Ki *measurements. To establish the optimal and safe dose range that would result in selective increase in BTB permeability without appreciably altering system blood pressure, various doses (0~120 μg/kg/min, i.v.) of NS1619 were administered in metastatic brain tumor bearing-nude rats. Additional experiments were performed by coinfusing of NS1619 with IBTX (1.0 μg/kg/min) to investigate whether inhibition of K_Ca _channels by IBTX has any effects on NS1619-induced permeability increase.

### Western Blot Analysis

The extracted protein samples were quantified to determine total protein concentrations using a protein assay kit (BioRad, CA). Same amount of each sample was fractionated on 10% SDS-polyacrylamide gel and then transferred to a nitrocellulose membrane. The membrane was probed with primary antibodies anti-MaxiKá (1:200; Santa Cruz Biotechnology, CA) and β-actin (1:5000; Sigma, MO), followed by peroxidase-conjugated secondary antibodies. The signals were detected with an enhanced chemiluminescence kit (Amersham Biosciences Corp., NJ). β-actin served as an internal control.

### Reverse Transcription-PCR

The extracted RNA was reverse transcripted using a Bioscript kit (Bioline) and Oligo (dT) _12–18 _primer (Invitrogene). The resulting cDNA products were used as templates for PCR assay. The genes of the K_Ca _channels were concurrently amplified with internal control β-actin in the same reaction tube as described previously [45]. Sequence specific primers were used for amplification of K_Ca _channels (forward: 5'-tccaaaacaaccaggctctc-3'; reverse: 5'- gggggagatgttgtgaagaa-3') and β-actin (forward: 5'- gcaccacaccttctacaatgagc-3'; reverse: 5'- ttgaaggtagtttcgtggatgcc-3'). PCR products were identified using agarose gel electrophoresis and ethidium bromide staining.

### Immunocytochemistry Staining

CRL-5904 cells and HBMEC were fixed with 4% paraformoldehyde for 15 min, and then incubated with anti-B2R (1:200; BD Bioscience, NJ) or anti-MaxiKα (1:200; Santa Cruz, CA) antibodies. The signals were detected with FITC-conjugated secondary antibodies (1:200; Jackson ImmnoResearch, CA). The cells were counterstained with 4', 6-diamidino-2-phenylindole (DAPI; Vector Laboratories, CA) and cover-slipped. Paraffin-embedded, metastatic brain tumor samples from lung cancer were deparaffinized and rehydrated. The slides were incubated with primary anti-MaxiKá and anti-B2R antibodies, and followed by biotinylated secondary antibodies (1:1000; Jackson ImmunoResearch, CA). Biotinylated conjugates were detected with avidin-biotin peroxide complex (Vector Laboratories), and then developed with 3, 3'-Diaminobenzidine (DAB) method. The sections were counterstained with hematoxylin. For the double staining, the sections were incubated with primary antibodies, anti-MaxiKá and anti-von Willebrand Factor (1:200; Chemicon, CA), and then subjected to FITC-and Tex-Red-conjugated secondary antibodies. The slides were examined under confocal microscopy. Negative control experiments were performed on all the corresponded specimens by deleting of primary antibodies.

## Competing interests

The author(s) declare that they have no competing interests.

## Authors' contributions

JH and KLB conceived the study. JH designed and performed all experiments. DY, MRS, XW, MKK, BMK, AE, KP participated in vivo BBB/BTB permeability assay. JH and XY drafted the original manuscript, and MKK, JMO, DI and KLB helped modify the final version of the manuscript.
